# Comparison of Three-Month HIIT and CMT Effects on Left Ventricle Echocardiography Observations in Male Employees

**DOI:** 10.3390/jcm11164795

**Published:** 2022-08-16

**Authors:** Hossein Shirvani, Maryam Moshkani Farahani, Mahmoud Ahmadzadeh, Alin Razvan Dinca

**Affiliations:** 1 Exercise Physiology Research Center, Life Style Institute, Baqiyatallah University of Medical Sciences, Tehran 1435916471, Iran; 2 Atherosclerosis Research Center, Baqiyatallah University of Medical Sciences, Tehran 1435916471, Iran; 3 Exercise Physiology, Department of Theory & Methodology of Team Sports, Faculty of Physical Education Kinetoterapie, and Recreation, State University of Physical Education and Sports, 2024 Chisinau, Moldova

**Keywords:** cardiovascular hemodynamics, end-systolic volumes, end-diastolic volumes, high-intensity interval training (HIIT), continuous moderate-intensity training (CMT)

## Abstract

The present study aimed to identify changes in echocardiographic parameters before and after three-month high-intensity interval training (HIIT) and continuous moderate-intensity training (CMT) in male employees. For this purpose, using a convenience sampling method, 33 male employees of the Islamic Republic of Iran Army (office workers with a sedentary lifestyle) aged 30 through 40 were selected. Participants were divided into three groups of HIIT, CMT, and control (11 for each group) including all anthropometric data (body fat percentage, body mass index, height, weight, and VO_2_ max) with no history of chronic diseases, metabolic syndrome, confirmed heart disease or congenital heart defect, and hospitalization due to chronic diseases or consumption of medication affecting cardiovascular indicators. A one-way ANOVA was conducted to compare the groups. The results demonstrated that the end-systolic volumes (ESVs) (*p* < 0.01) and relative wall thickness (RWT) in the CMT group (*p* < 0.01) and the end-diastolic volumes (EDVs) (*p* < 0.01), stroke volumes (SVs) (*p* < 0.01), end-systolic and diastolic diameters (ESD, EDD) (*p* < 0.01), as well as the RWT and left ventricle diastolic function (E/A ratio) in the HIIT group (*p* < 0.05) were significantly different before and after the 12-week training (Bonferroni correction was used for pairwise comparisons). The results revealed a significant increase in the end-systolic diameters (ESDs) of the HIIT group, whereas no such increase was observed in the ESDs of the CMT group (*p* < 0.51). Moreover, a significant increase was observed in left ventricular (LV) RWT and aerobic power of both training groups. The significant decrease of ESVs and the significant increase in E/A ratio, ESDs, EDDs following HIIT (two to three sessions per week) may indicate beneficial and optimal LV structural adaptations and improved LV function in nonathletes (even with a sedentary lifestyle).

## 1. Introduction

Exercise physiology researchers, who pursue the goal of improving cardiovascular health through exercise, have conducted numerous exhaustive research projects based on the two primary variables of exercise, i.e., intensity and duration [1]. The problem with these projects lies in their implementation from a temporal perspective, whose training regimens normally extend from 3 to 12 weeks [1,2,3]. Considering the effective mechanism of exercise-related stress and the training variables of exercise on the cardiac physiological-hemodynamic system, the minimum period for observing changes is over 12 weeks [1]. Nevertheless, certain hemodynamic changes can be evident following the first week of training. Several research studies have shown that physical activities and exercise can stabilize the physiological effects of stress [1,2,3,4]. In a military work environment, regular exercise training may elicit employees’ satisfaction and, consequently, a greater efficiency from an aspect of physical and mental health in office working positions, which demand a higher level of stress-controlling abilities. In parallel with improved stress management due to regular exercise training, physiological effects of various exercise training regimens, in general, and cardiovascular effects of such regimens, specifically, have recently been interesting fields of study for exercise physiologists and cardiologists. Of the various types of training regimens, continuous moderate training (CMT) and high-intensity interval training (HIIT) have always been the most debated topics considering their efficacy on cardiovascular and cardiac function [1]. HIIT is a high-intensity (greater than or equal to 85% of the heart rate maximum (HRmax)) interval training with short recovery periods, whereas MCT normally consists of exercises between 60 and 85% of HRmax. Previous evidence suggests that both HIIT and CMT improve physical and psychological outcomes, anxiety, or depression, although adherence to physical exercise and cardiovascular improvements seems to be greater in HIIT [5,6,7]. 

Comparing the cardiac muscle functional changes in endurance athletes and a control group using Doppler ultrasound and echocardiography, Douglas et al. observed a normal systolic function in endurance athletes as well as in the control group (nonathletes) [8]. Similarly, Brandao et al. failed to demonstrate a significant difference in the LV contraction capacities of male athletes and an inactive control group (15 min of high intensity interval training for seven sessions) [9]. Sadaniantz et al. evaluated inactive elderly males before and after a yearlong training program (cycling, 4 h per week) [10]. The echocardiographic measurements of ejection fraction (EF) and fractional shortening (FS) did not reveal any significant difference in the LV systolic function. In their macroscale meta-analysis, Harris et al. reported no significant difference between endurance athletes and control group in terms of the echocardiographic indicators of FS, EF, and LV as was found in Pluim et al. research [4,11,12]. Spina et al. examined the LV contraction function in response to the β-adrenergic stimulation of cardiac muscle in 10 females and 6 males before and after a 12-week cycling and running exercise program. Changes in FS were significantly greater than in the pretraining period, implying an enhanced hemodynamic values and LV contraction function, but considering the HIIT strategy for adaptations in the study conducted by Gibala et al. was demonstrated merely for skeletal muscle [13,14]. Baggish et al. performed a longitudinal analysis of the training of 40 endurance athletes, in which the diastolic function improved following a 90-day training program. Although data on the indicators of diastolic filling rate at rest are ambiguous, strong evidence suggests a considerable improvement in the LV diastolic function as a result of continuous physical activity in young and elderly athletes [15]. The effects of high-intensity interval training (HIIT) on myocardial adaptations in nonhuman specimens have been examined in multiple studies but few studies specifically have aimed for structural adaptations of left ventricle following HIIT training [12,16,17,18].

The different pattern of LV hypertrophy in trained athletes was first demonstrated. Despite the abundance of studies reporting such hypertrophic differences, researchers must not view these adaptations as definitive or binary phenomena; rather, they should be regarded as relative hypotheses (notions). Physical activities and exercises are rarely either purely static or dynamic, with training programs overlapping in the majority of cases. Therefore, structural adaptations of the cardiac system arising from endurance training are the result of volume–load combinations. The evident benefits and effects of HIIT regimens compared to CMT on left ventricle function and echocardiography parameters have not clearly been revealed and identified [19,20].

Echocardiography has become a suitable alternative technique owing to its relatively low cost and short duration, making it possible to conveniently examine the cardiovascular status of the administrative staff of the Islamic Republic of Iran Army (office workers) as an initial step [21]. Through an echocardiographic evaluation, it is possible to identify any potentially desirable or undesirable changes in the cardiovascular system that may possibly increase left ventricle function and hemodynamic values, or that may otherwise increase the risk of sudden death or reduce the risk of developing cardiovascular disorders as a result of training programs [4,17]. Accordingly, the present study aimed to identify changes in hemodynamic indicators, including end-diastolic volume (EDV), end-systolic volume (ESV) and end-systolic diameter (ESD), end-diastolic diameter (EDD), relative wall thickness of the left ventricle (RWT) following 12-week HIIT and CMT program regimens as well as left ventricle function (E/A ratio).

## 2. Materials and Methods

A pretest–post-test design was used to conduct this research. The study was conducted according to the guidelines of the Declaration of Helsinki and approved by the Ethics Committee of Baqiyatallah University of Medical Science with protocol code: IR.BMSU.REC.1398.002 and approval date 16 April 2019.

Participants were included for the present research from a convenience sampling method. For this purpose, 33 male employees of the Islamic Republic of Iran Army (office workers with a sedentary lifestyle) aged 30 through 40 were selected and they gave their informed consent. The participants were divided into three groups: HIIT, CMT, and control (11 participants for each group). Participants underwent an echocardiography examination at Dr. Soleimani Echocardiography Clinic, Tabriz, Iran. The majority of echocardiography parameters including EDV, ESV, ESV, EDD, ESD as well as RWT and E/A ratio, were measured by an experienced echocardiologist during an examination (as well as anthropometric data collection). This procedure was carried out twice, before and after the 12-week training program.

Thirty-two training sessions were designed in a way that allowed two sessions per week in the first 4 weeks and three sessions per week in last 8 weeks. Assessments were performed using a transducer whereby high-frequency sound waves were transmitted to the heart. According to recommended standards, the parasternal long-axis view was used to measure the left ventricular internal diameter and wall. Values were carefully measured perpendicular to the LV longitudinal axis immediately below the level of the mitral valve leaflet tips.

The proposed CMT and HIIT regimens were performed while cycling on an ergometer (ERGO-FIT Ergometer Cardio Line 407 Med). The HIIT group was given a 1:4 work:rest with active recovery (3.7 to 3.9 W·kg^−1^ interspersed by active recovery with 1.2 to 1.4 watts·kg^−1^). The CMT group was given a continuous moderate training with 2.0 to 2.4 watts·kg^−1^ from 30 min in a session to 35 min with 2 sessions a week in the first phase and 3 sessions a week in the late phase of training. All participants were evaluated through echocardiography and medical ultrasound to measure all dependent variables. After the initial evaluation in the pretest phase, participants began their respective trainings depending on the group to which they were assigned, i.e., control, HIIT, and CMT (Table 1).

The HIIT and CMT programs were designed in 32 sessions at a particular time of the day to avoid affecting the circadian rhythm of the heart rate and blood pressure. It should be noted that the training loads and volumes were matched from the aspect of the energy expenditure of the two training types (calories burned in HIIT were calculated at different load intensities (in watt and based on the heart rate) and at an identical speed to the calories burned in CMT) [18,22]. The HIIT and CMT programs were implemented by designing 8 training microcycles for each, to match the energy expenditure and the calories burned during the 32 training sessions. Warm-up and cool-down lasted for 10 min in both training groups [19,23,24].

Having completed the training protocols with a 2-day break (to regain body fluid balance and reverse the acute effects of training, 24–48 h after the last training session), participants underwent post-test echocardiography. The obtained data were analyzed using a repeated measures ANOVA at a significance level of less than 0.05 (*p* < 0.05). All data collected were analyzed in IBM SPSS Statistics for Windows, version 22.0 (Tabriz, Iran) after examining the normal distribution of data using the Shapiro–Wilk test and the homogeneity of variances using a one-way ANOVA.

## 3. Results 

### 3.1. Ventricular Volumes

In this research, 33 inactive, healthy, and conveniently accessible male participants were assigned to three groups, i.e., control, CMT, and HIIT, and underwent an echocardiographic evaluation in two periods of time. The normality of the data was evaluated using a Shapiro–Wilk test and there were no significant differences in pretest data distribution between the three groups in terms of the variables measured (*p* > 0.05) (Table 1).

The analysis of the homogeneity of variances and normality of distribution using the one-way ANOVA and Shapiro–Wilk test in terms of the ESVs, EDVs, and SVs before the 12-week training program (*p* = 0.01, F = 4.7; *p* < 0.01, F = 7.4; and *p* < 0.01, F = 7.9, respectively) are shown in Table 2.

**Figure 1:**
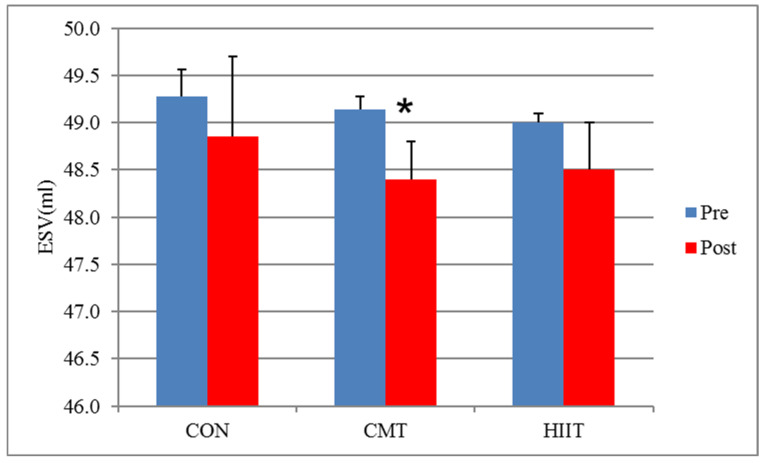
ESV Changes before and after 12 Weeks of CMT and HIIT; * Indicates a significant decrease compared with the HIIT and control groups (*p* < 0.05). ESV: End Systolic Volume (mL). CON: Control. CMT: Continuous Moderate Training. HIIT: High Intensity Interval Training.

**Figure 2:**
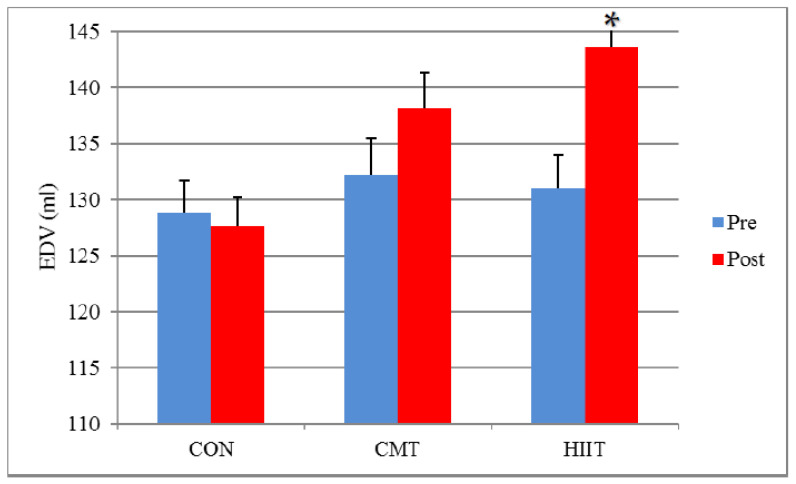
EDV Changes before and after 12 Weeks of CMT and HIIT; * Indicates a significant increase compared with the CMT and control groups (*p* < 0.05). EDV: End-Diastolic Volume (mL). CON: Control. CMT: Continuous Moderate Training. HIIT: High-Intensity Interval Training.

**Figure 3:**
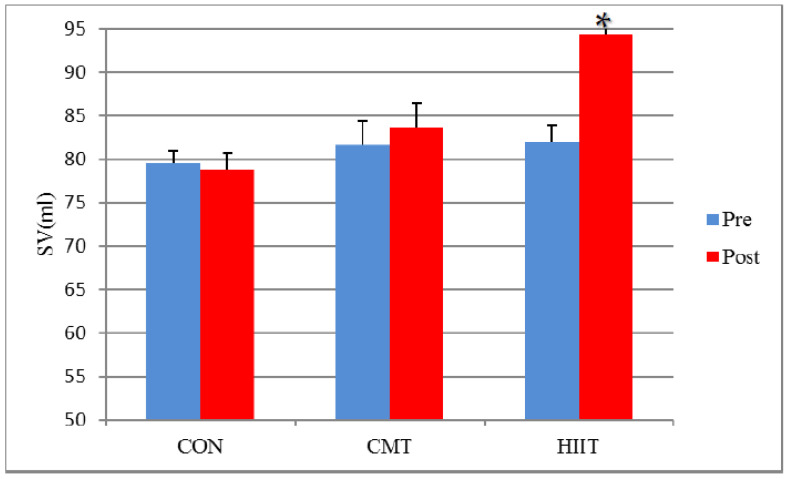
SV Changes before and after 12 Weeks of CMT and HIIT; * Indicates a significant increase compared with the CMT and control groups (*p* < 0.05). SV: Stroke Volume (mL). CON: Control. CMT: Continuous Moderate Training. HIIT: High-Intensity Interval Training.

Results from the Bonferroni correction for the pairwise comparison of the study groups revealed significant differences in the ESVs (*p* < 0.05) and RWT (*p* < 0.01) of the CMT and control groups before and after the 12-week program. The ESVs reduced significantly in the CMT group compared with the control group before and after the 12-week program (Figure 1). Moreover, results from the Bonferroni correction demonstrated no significant difference in the EDVs and SVs of the CMT and control groups before and after the 12-week program (Figure 2 and Figure 3). Additionally, the RWT of the CMT group showed a significant increase compared with that of control group following the 12-week program (*p* < 0.01).

Moreover, results demonstrated no significant difference in the EDVs and SVs of the CMT and control groups before and after the 12-week program (Figure 2 and Figure 3). Additionally, the RWT of CMT group showed a significant increase compared with that of the control group following the 12-week program (*p* < 0.01).

The results did not indicate any significant difference in the ESVs of the HIIT and control groups before and after the 12-week program (*p* > 0.05). Results showed a significant increase in the EDVs and SVs of the HIIT and control groups before and after the 12-week program (EDVs (*p* < 0.01) and SVs (*p* < 0.01)).

There was a significant difference between the HIIT and CMT groups in terms of ESVs (decreased in CMT), EDVs (increased in HIIT), and SVs (increased in HIIT) before and after the 12-week program (*p* = 0.01, respectively) (Figure 1, Figure 2 and Figure 3). There was a significant increase in the EDVs (*p* < 0.01) and SVs (*p* < 0.01) of the HIIT group compared with the those of the CMT group after the 12-week training period (Figure 2 and Figure 3). 

### 3.2. Ventricular Diameters and Relative Wall Thicknesses

#### 3.2.1. End-Diastolic Diameter (EDD)

Results from the pairwise comparison of the study groups did not demonstrate any significant difference in the LV EDDs of the CMT group compared with those of the control group before and after 12 weeks of training (*p* > 0.05) (Figure 3). 

**Figure 4:**
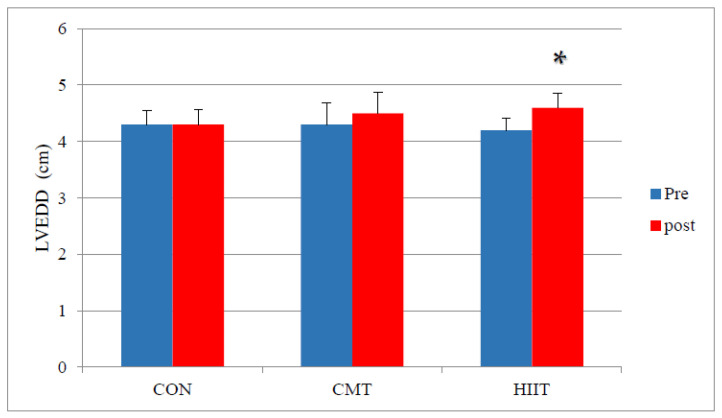
Mean EDD Changes (cm) before and after 12 Weeks of CMT and HIIT; * Indicates a significant increase compared with the CMT and control groups (*p* < 0.05). LVEDD: Left Ventricle End-Diastolic Diameter (cm). CON: Control. CMT: Continuous Moderate Training. HIIT: High-Intensity Interval Training.

Results revealed a significant increase in the LV EDDs of the HIIT group compared with those of the control group before and after 12 weeks of training (*p* = 0.03) (Figure 4). Similarly, the results showed a significant increase in the LV EDDs of the HIIT group compared with those of the CMT group before and after the 12-week training period (*p* = 0.04). Examining the effect size of the HIIT group through the partial eta-squared method, it was observed that 49% of the variance in the increase of EDDs was explained by HIIT (Figure 4).

#### 3.2.2. End-Systolic Diameter (ESD)

**Figure 5:**
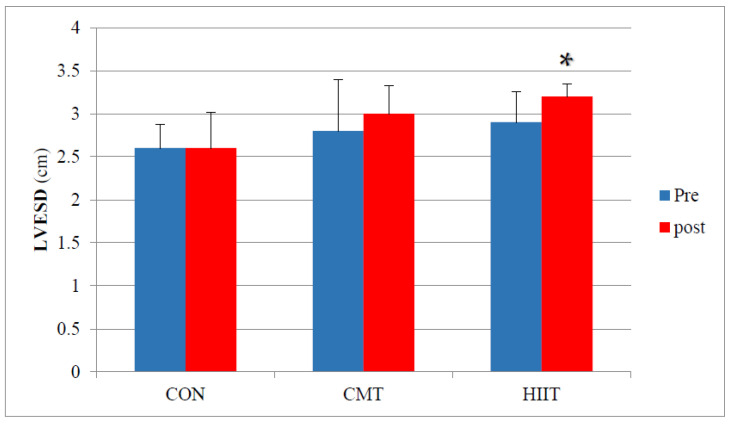
Mean ESD Changes (cm) before and after 12 Weeks of CMT and HIIT; * Indicates a significant increase compared with the HIIT and control groups (*p* < 0.05). LVESD: Left Ventricle End-Systolic Diameter (cm). CON: Control. CMT: Continuous Moderate Training. HIIT: High-Intensity Interval Training.

Results from pairwise comparison of the study groups did not reveal a significant increase in the LV ESDs of the CMT group compared with those of the control group before and after 12 weeks of training (*p* = 0.08) (Figure 5). Results of this research demonstrated a significant increase in the LVESDs of the HIIT group compared with those of the control group before and 12 weeks after the research design (*p* = 0.01). Similarly, the results showed a significant increase in the LVESDs of the HIIT group compared with of those of the CMT group before and 12 weeks after the research design (*p* = 0.02). Examining the effect size of the CMT group through the partial eta-squared method, it was observed that 43% of the variance in the increase of ESDs was explained by CMT (Figure 5).

### 3.3. Ventricular Relative Wall Thicknesses (RWT)

**Figure 6:**
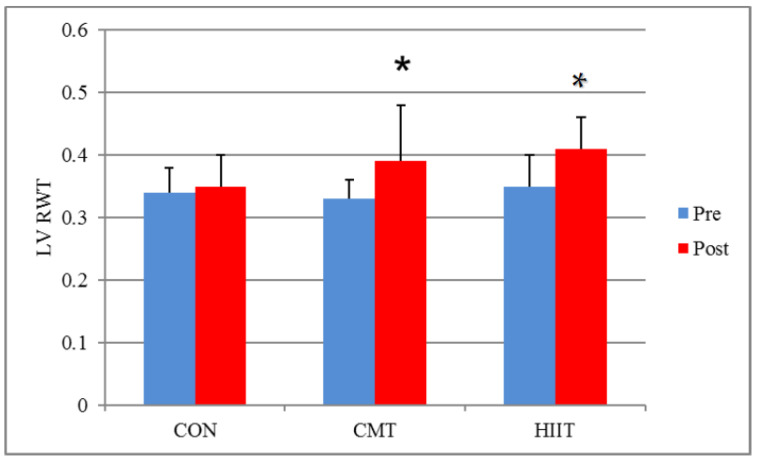
Mean LV RWT Changes before and after 12 Weeks of CMT and HIIT; * Indicates a significant increase compared with the control group (*p* < 0.05). LV RWT: Left Ventricle Relative Wall Thickness. CON: Control. CMT: Continuous Moderate Training. HIIT: High-Intensity Interval Training.

Results from the Bonferroni correction for the pairwise comparison of the study groups showed a significant difference in the LV RWT of the CMT and HIIT groups compared with that of the control group before and after 12 weeks of training, where the former groups revealed a significant increase in the specified values compared to the latter (*p* = 0.03) (Figure 6). Additionally, the results demonstrated a significant difference between the HIIT and control groups in terms of LV RWTs before and after 12 weeks of training, where the former group witnessed a significant increase in the specified values compared to the latter (*p* = 0.04) (Figure 6). Similarly, the results did not show any significant difference between the LV RWTs of the HIIT and CMT groups before and after 12 weeks of training (*p* > 0.05). Examining the effect size of the HIIT and CMT groups through the partial eta-squared method, it was observed that 36% and 30% of the variance in the increase of LV RWTs could be explained by the HIIT and CMT, respectively (Figure 6). 

### 3.4. Left Ventricular Function (E/A Ratio)

**Figure 7:**
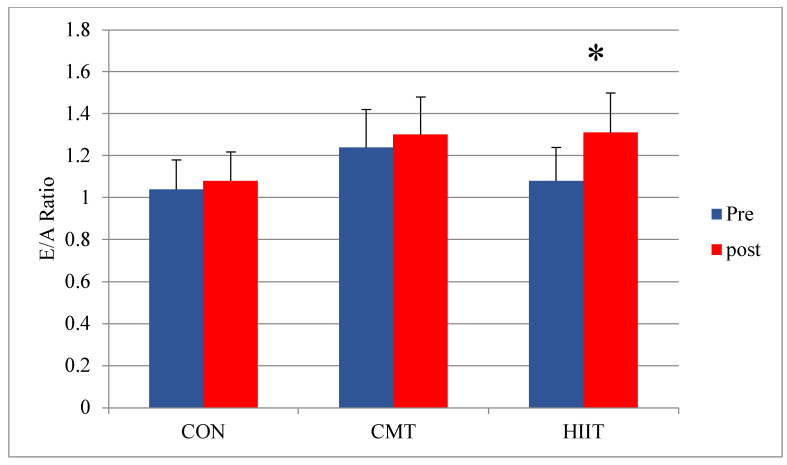
Mean LV E/A Ratio (Left Ventricle Function) Changes before and after 12 Weeks of CMT and HIIT; * Indicates a significant increase compared with the control group (*p* < 0.05). E/S Ratio: Early Filling Rate Divided by Secondary Filling Rate (Left Ventricle Function). CON: Control. CMT: Continuous Moderate Training. HIIT: High-Intensity Interval Training.

Results from the pairwise comparison of the study groups revealed a significant increase in the E/A ratio (as an indicator of left ventricle function) of the HIIT group compared with that of the control group before and after 12 weeks of training (*p* = 0.01) (Figure 7). The ratio of the early filling rate due to the peak velocity blood flow from the left ventricular relaxation in early diastole (E wave) to the peak velocity flow in late diastole caused by atrial contraction (the A wave) is an indicator of left ventricle function. 

## 4. Discussion

The different, and occasionally contradictory, cardiovascular responses to various HIIT and CMT regimens have attracted the attention of exercise physiology researchers. Accordingly, the present study aimed to compare the effects of 3 months of HIIT with those of CMT on the cardiovascular hemodynamics (echocardiography) of employees (office workers with sedentary lifestyle). Based on the results of the present study, there were a significant decreased SV in the CMT group and increased EDV and SV in the HIIT group when compared with the control group but not between the CMT and HIIT groups. The estimation of hemodynamic changes in this study may have been hindered by factors such as the examination of the fluid intake status and the measurement of the plasma volume or blood volume changes, complicating a more favorable evaluation of ESV, EDV, and SV across the CMT and HIIT groups.

The specified results are inconsistent with those obtained by Green et al. [25] who used the term “Cardiac Fatigue” to refer to the effect of long-term high-intensity interval training. Furthermore, they observed significant heterogeneous changes in ESV, EDV, SV and other morphological indicators such as ventricular mass and LV RWT, whereas the results of the present study provided no evidence of cardiac fatigue or heterogeneous changes in ESV, EDV, and SV although the RWT in both groups may indicate heterogeneous changes; however, additional studies are needed to clearly confirm them. 

Given the necessity of controlling training in terms of load, intensity and number of sessions, another possible reason for the heterogeneous cardiovascular changes as a result of HIIT or any other form of high-intensity training appears to be that they are beneficial to induce desired changes in cardiac function. A systematic periodization and design of training sessions can lead to health-enhancing cardiovascular responses with the including two to three sessions HIIT training per week which has been recommended by Volpe et al. [26]. Considering cardiac hypertrophy, it was concluded that the left ventricle was enlarged nonpathologically as a result of HIIT (clearly evident due to increased EDD, ESD, RWT, and E/A ratio) and CMT (not clearly evident due to the change in RWT and but not in left ventricle diameters) without causing any pathological symptom, so that it can be distinguished from the athlete’s heart. Training duration is another factor that needs to be taken into account. In order to arrive at more accurate conclusions and ensure the initiation of the signaling process leading to anatomical modifications, the training duration is scheduled for a minimum of three months in the majority of studies. Due to limitations in accessing facilities, the training duration of the present research was scheduled for 12 weeks. 

Marja et al. [27] examined the effect of two weeks of HIIT and CMT on diabetic patients and saw an increase in their EDVs, ESVs, and COs as a result of both types of training. These findings are inconsistent with those of the present study, which may be attributable to the dissimilar clinical status of participants, state-of-the-art equipment for the measurement of indicators, and the larger number of participants (twice as large) in their study compared to ours. Their research protocol offered a scientific overview, involving 4–6 30 s cycling sets in laboratory conditions until failure.

Important points can be derived regarding the changes in LV ESDs, LV EDDs, and E/A ratio (LV function) following the 12-week HIIT training in the present study. The increase of LV EDDs in the HIIT group compared with the CMT group signifies the increase of the venous return (VR) to the heart. Increased VRs in accordance with the Frank–Starling law increase the stretch on the LV muscle fibers and, consequently, the LV EDD. HIIT regimens may contribute to a greater angiogenesis and increase the early LV filling time (E wave) in the HIIT group due to a greater cardiac rest time as a result of increased EDD and reduced resting heart rate following training, thus improving LV function. A greater cardiac rest time can ultimately reduce myocardial workload. The HIIT approach can be used to serve a protective role in reducing myocardial workload in inactive individuals or those at risk for heart disease. The significant increase in EDDs and less increase in ESDs of the HIIT group are worth noting, as they are inconsistent with the findings of Scharf et al. [28], who provided 16 weeks of HIIT and CMT to inactive male participants and discovered a significant increase in the LV internal diameter (systolic and diastolic) as well as in the LV mass index as a result of HIIT. Their training protocol consisted of running and walking on a treadmill, which was one of the limitations of their study in that HIIT and CMT had not been offered under isocaloric conditions. The training intensity during the 16-week period should be controlled by determining VO_2_ max and the maximum heart rate to apply an accurate intensity and allow its efficiency to be examined through retest [29]. In addition, the training intensity in the study from Scharf et al. was greater than that of the present research (calculated based on the maximum heart rate). The inconsistencies in the findings of the two studies may be accounted for by HIIT intensity differences and the different training protocols and durations (nearly twice as long) [28].

A number of notable points can also be elucidated concerning LV RWTs. The primary causes of LV hypertrophy are the enhancement of hemodynamic indicators and the activation of the renin–angiotensin pathway, which activates the AT1 receptors and increases the RWT [30]. It is worth noting that no significant difference was observed between HIIT and CMT in terms of an LV RWT increase, with both types of training having a similar contribution to such an increase. Nevertheless, the greater role of HIIT in increasing the LV RWT has been reported in the majority of studies, including that conducted by Huang [31], who identified a slight increase in LV RWT and LV hypertrophy following HIIT as opposed to CMT. The small number of accessible participants in that study may have contributed to a lack of significant distinction between the HIIT and CMT groups.

## 5. Conclusions

The results of the present study demonstrated significant differences in hemodynamic indicators (ESV, EDV, SV, LVEDD, LVESD, RWT, and E/A ratio) of the heart as a result of HIIT and CMT although the hemodynamic changes were small in the latter. The estimation of hemodynamic considerable changes (EDV, SV) in CMT may have been hindered by factors such as the examination of the fluid intake status and the measurement of the plasma volume or blood volume changes, complicating a more favorable evaluation of the ESV, EDV, SV, LVEDD, LVESD, RWT, and E/A ratio as indicators of left ventricle function. However, these changes were significantly obvious in the HIIT regimen. It is recommended that cross-sectional echocardiographic studies be performed on LV structural changes in physically trained and untrained individuals.

## Figures and Tables

**Table 1:** Mean and Standard Deviation of Ventricular Volumes and Primary and Secondary Filling Rates.

Variable Group	EDV (mL)	ESV (mL)	Ventricular Mass Index (g m^2^)	SV (mL beat)	Primary Filling Rate (E) (mL/s)	Secondary Filling Rate (A) (mL/s)	Primary and Secondary Filling Ratio
**Control**	128.4 ± 8.85	49.1 ± 2.86	75.8 ± 4.3	79.3 ± 57.2	65.28 ± 2.69	63.28 ± 2.69	1.04 ± 0.15
**CMT**	132.2 ± 20.50	49.0 ± 14.94	79.5 ± 7.87	81.1 ± 71.81	77.5 ± 8.8	64.00 ± 3.03	1.24 ± 0.19
**HIIT**	131.2 ± 00.37	1.01 ± 00.49	77.5 ± 8.2	82.1 ± 00.44	80.00 ± 9.6	65.70 ± 1.87	1.08 ± 0.42
**Shapiro–Wilk**	*p* > 0.05	*p* > 0.05	*p* > 0.05	*p* > 0.05	*p* > 0.05	*p* > 0.05	*p* > 0.05

CMT: Continuous Moderate Training. HIIT: High-Intensity Interval Training. EDV: End-Diastolic Volume. ESV: End-Systolic Volume. SV: Stroke Volume. E: Primary Filling Rate. A: Secondary Filling Rate.

**Table 2:** Mean ± SD of Weight, Height, Body Fat Percentage, and Aerobic Power of Participants before Training.

Variable Group	Weight (kg)	Height (cm)	VO_2_ Max (mL/kg/min)	Fat Percentage	BMI (kg/m^2^)	EDD (mm)	ESD (mm)	RWT
Control	78.6 ± 2.1	175.5 ± 1.8	43.5 ± 2.3	21%	25.5	6.4 ± 0.08	2.63 ± 0.3	0.34 ± 0.1
CMT	78.5 ± 1.8	173.7 ± 2.81	42.7 ± 1.8	23%	26	5.4 ± 0.12	2.65 ± 0.7	0.33 ± 0.08
HIIT	78.5 ± 2.2	175 ± 2.5	42.8 ± 2.2	21%	25.6	5.4 ± 0.11	2.9 ± 0.3	0.35 ± 0.12
Shapiro–Wilk	*p* = 0.88	*p* = 0.11	*p* = 0.35	*p* = 0.18	*p* = 0.32	*p* = 0.08	*p* > 0.1	*p* > 0.12

CMT: Continuous Moderate Training. HIIT: High-Intensity Interval Training. BMI: Body Mass Index. EDD: End-Diastolic Diameter. ESD: End-Systolic Diameter. RWT: Relative Wall Thickness.

## Data Availability

All data are available on request from the authors.

## Abbreviations

HIIT	High-intensity interval training	
CMT	Continuous moderate-intensity training	
ESVs	End-systolic volumes	
EDVs	End-diastolic volumes	
RWT	Relative wall thickness	
ESDs	End-systolic diameters	
EDDs	End-diastolic diameters	
E/A	Ratio of early filling rate (E wave) to secondary filling rate (A wave)	
BMI	Body mass index	
LV	Left ventricular	
VO_2_ max	Maximal oxygen consumption	
